# Serum Biomarkers of Inflammation and Turnover of Joint Cartilage Can Help Differentiate Psoriatic Arthritis (PsA) Patients from Osteoarthritis (OA) Patients

**DOI:** 10.3390/diagnostics11010052

**Published:** 2020-12-31

**Authors:** Michał Waszczykowski, Anna Fabiś-Strobin, Igor Bednarski, Aleksandra Lesiak, Joanna Narbutt, Jarosław Fabiś

**Affiliations:** 1 Department of Arthroscopy, Minimally Invasive Surgery and Sports Traumatology, Medical University of Lodz, Kosciuszki 4, 90-419 Lodz, Poland; jaroslaw.fabis@umed.lodz.pl; 2 Clinic of Orthopaedic and Traumatology, Polish Mother’s Memorial Hospital Research Institute, Rzgowska 281/289, 93-338 Lodz, Poland; anna.m.fabis@gmail.com; 3 Dermatology, Pediatric Dermatology and Oncology Clinic, Medical University of Lodz, Kosciuszki 4, 90-419 Lodz, Poland; igorbednarskiv@gmail.com (I.B.); aleksandra.lesiak@umed.lodz.pl (A.L.); joanna.narbutt@umed.lodz.pl (J.N.)

**Keywords:** osteoarthritis, psoriatic arthritis, aggrecan, COMP, biomarkers, serum, overlapping

## Abstract

The aim of this study was to find characteristic biomarkers in the serum of patients with osteoarthritis (OA) and psoriatic arthritis (PsA) responsible for inflammation and destruction of joint cartilage, which could differentiate these two diseases. The study included 67 people: 22 patients with knee OA, 22 patients with PsA, and 23 individuals who were the control group of healthy individuals (HC). The concentration of IL-18, IL-20, IL-6, MMP-1, MMP-3, COMP, PG-AG, and YKL-40 in serum were determined. Among the OA and PsA patients group, the radiological assessment and clinical assessment were also performed. The concentration of 7 out of 8 of examined biomarkers (except MMP-1) was statistically significantly higher in the serum of patients with OA and PsA than in the control group. Compering OA and PsA groups only, the serum PG-AG level in OA patients was statistically significantly higher than in PsA patients (*p* < 0.001). The results of univariate and multivariate logistic regression analysis comparing OA and PsA biomarker serum levels identified PG-AG and COMP as markers that are significantly different between patients with OA and PsA (odds ratio 0.995 and 1.003, respectively). The ROC curve constructed using the model with age showed PG-AG and COMP had an AUC of 0.907. The results of this study show that COMP and PG-AG may be sensitive markers differentiating patients with osteoarthiritis from psoriatic arthritis.

## 1. Introduction

Osteoarthritis (OA) is a progressive degenerative process occurring within the joint cartilage, leading to its slow destruction and dysfunction of the involved joints [1,2,3]. Psoriatic arthritis (PsA), on the other hand, is a seronegative inflammatory arthropathy manifesting itself in skin lesions and progressive inflammatory changes within the musculoskeletal system, leading to its gradual dysfunction [4,5,6]. Therefore, it seems that, in the basic approach, these are two different disease entities with different etiopathology, course, and clinical conditions, but similar consequences. In PsA, inflammatory processes dominate, which concern periarticular tissues: entheses amd ligaments [4,5,6,7]. In OA, the changes are primarily degenerative, manifesting themselves mainly in the articular cartilage and subchondral bone, gradually leading to joint dysfunction [1,2,3]. In PsA, joint changes are usually asymmetric and affect several joints [4,5,8]. In OA, the degenerative changes affect one large joint, several large joints, or the spine [1,2,3]. If there are typical changes in PsA, which are additionally accompanied by characteristic skin lesions (plaque psoriasis) and changes on the nails (subungual hypekeratosis, pitting), then a proper diagnosis and effective treatment implementation are clear and reasonable [8]. All of this together prevents severe and irreversible changes in the musculoskeletal system. Similarly, in an OA, if it has a typical sign and course, the diagnosis and management for the therapeutic team is not problematic. However, in OA, there are patients who manifest similar symptoms like PsA patients [9]. Sometimes patients with osteoarthritis present symptoms of inflammation: entezopathies, tendinopathies, and joints swelling [8,9,10,11]. At other times, patients with PsA show clinical symptoms that are more indicative of changes in the joint itself and cartilage destruction [9,10,11]. Moreover, the location of dominant lesions and symptoms in both diseases might be similar [9,12]. Degenerative changes and pain in OA are often located in the cervical spine, which also affects some patients with PsA [2,3,13]. A similar situation exists with the peripheral form and symmetric changes in PsA, where the interphalangeal joints of the hands are mostly involved [4,9]. A similar manifestation of these changes can also be found in patients with OA [12]. This specific pattern of overlap between the two disease entities may delay proper diagnosis and implementation of effective treatment [14].

There is still little information in the literature on the differentiation of OA and PsA based on specific serum biomarkers. Therefore, it seems that the identification of specific biomarkers for these diseases may be helpful in their early diagnosis and appropriate treatment.

The aim of this study was, therefore, to find characteristic biomarkers in the serum of patients with OA and PsA responsible for inflammation and destruction of joint cartilage, which could differentiate the two diseases and their concentration in the serum would be disease-specific. The concentration of biomarkers responsible for inflammation and destruction of joint cartilage were analysed in the serum of PsA patients, OA patients, and the control group of healthy individuals (HC): interleukin 6 (IL-6), interleukin 18 (IL-18), interleukin 20 (IL-20), matrix metalloproteinases 1 and 3 (MMP-1, MMP-3), cartilage oligomeric matrix protein (COMP), human cartilage glycoprotein (YKL-40), and aggrecan (PG-AG). Our earlier studies and the others confirm the important role these biomarkers may play in the etiopathogenesis of both OA and PsA [15,16,17,18,19,20,21,22]. Determination of the clinical stage of disease expressed on a Kellgren-Lawrence scale, Western Ontario and McMaster Universities Osteoarthritis Index (WOMAC), Index of Severity for Osteoarthritis of the Knee (Lequesne index) for OA patients and Body Surface Area score (BSA), Psoriasis Area and Severity Index (PASI), Dermatology Life Quality Index (DLQI), and the number of painful (TJC) and swollen joints (SJC) for PsA patients was also conducted [23,24,25,26,27,28,29,30,31,32].

## 2. Materials and Methods

Sixty-seven people were enrolled in the study including 22 patients with OA, 22 patients with PsA, and 23 individuals who were the control group of healthy individuals (HC). In the OA group, based on clinical examination and the American College of Rheumatology criteria, the diagnosis of osteoarthritis of the knee joint was made [33]. The main inclusion criteria in this group were the clinical and radiological changes of OA of the knee in 2nd, 3rd, and 4th stage of the Kellgren-Lawrence scale [27]. The main exclusion criteria in this group were history of immunological diseases (rheumatoid arthritis—RA, PsA, Crohn’s disease), neoplastic diseases, and recent or past significant knee joint injuries. In the PsA group, based on the clinical examination and the Classification of the Psoriatic Arthritis Study Group (CASPAR) criteria, the diagnosis of psoriatic arthritis was made [34]. All the PsA patients presented simultaneously with moderate plaque psoriasis. Most PsA patients (80%) had an asymmetric type of the disease and involvement of few joints. In 45% of them, nail changes (subungual hyperkeratosis, pitting) were observed. In 20% of the patients, we diagnosed a symmetrical form of PsA with nails involvement in all the cases. The main exclusion criteria in this group were: a history of past or present immunological diseases (RA, Crohn’s disease), neoplastic diseases, and present or recent (6 months before) anti-TNFα treatment.

The patients were qualified for the study at the Orthopaedic Outpatient Clinic, Department of Arthroscopy, Minimal Invasive Surgery and Sport Traumatology (OA group) before the knee replacement surgery or other arthroscopic cartilage repair and at the Department of Dermatology and Venereology (PsA group).

The control group consisted of persons corresponding to age and gender distribution for the study group, in whom no clinical and radiological symptoms of degenerative disease nor features of psoriasis and arthritis were found and no other criteria were found to exclude them from the study.

All patients and control group members gave their informed and written consent to participate in the study. The study was conducted in accordance with the Declaration of Helsinki, and the protocol was approved by the local Bioethics Committee of the Medical University of Lodz, Lodz, Poland (consent no.: RNN/36/06/KB; 21.02.2006).

### 2.1. Clinical Assessment OA Group

The clinical evaluation of patients and the stage of disease progression was based on the Western Ontario and McMaster Universities Osteoarthritis Index (WOMAC) and LEQUESNE Index of Severity for Osteoarthritis of the Knee [23,24,25,26]. On the basis of radiological examinations, the degree of radiological advancement of the knee osteoarthritis was assessed using the criteria described by Kellgren I Lawrence [27].

### 2.2. Clinical Assessment PsA Group

The Body Surface Aresa score (BSA), Psoriasis Area and Severity Index (PASI), and Dermatology Life Quality Index (DLQI) as well as 68 tender and 66 swollen joint count (TJC, SJC) were used in the clinical assessment of patients and the severity of the disease [28,29,30,31,32].

### 2.3. Determination of the Biomarker Levels of Inflammation and Cartilage Turnover in Serum

Subsequently, IL-6, IL-18, IL-20, MMP-1, MMP-3, COMP, YKL-40, and PG-AG serum concentration measurements were performed with enzyme-linked immunosorbent assay (ELISA) kits in the OA and PsA study group as well as a HC (healthy control) group. Peripheral blood samples were collected from each patient in the morning, then centrifuged, and serum samples were collected to 1.5-mL eppendorfs, which were then sealed, frozen, and stored at −80 °C for further immuno-enzymatic testing. Serum activity measurements were performed with enzyme-linked immunosorbent assay (ELISA) kits from R&D Systems Europe, Ltd, Abingdon, UK (IL-6, IL-18, IL-20, MMP-1, MMP-3), BioVendor GmbH, Heidelberg, Germany (COMP), Metra Quidel, San Diego, CA, USA (YKL-40), BioSource Europe S.A., Nivelles, Belgium (PG-EASIA), according to the manufacturers’ instructions. The minimum detection level has been determined: 0.7 pg/mL for IL-6, 4.57 pg/mL for IL-18, 16.6 pg/mL for IL-20, 0.095 ng/mL for MMP-1, 0.045 ng/mL for MMP-3, 0.4 ng/mL for COMP, 10 ng/mL for YKL-40, and 0.9 ng/mL for PG-AG. The same activities (blood collection, centrifugation, freezing, and storage) were performed in the control group, which was followed by the same immuno-enzymatic assays as in the study groups using appropriate ELISA test kits (R&D Systems, BioVendor, Metra Quidel, BioSource). Serum samples in the OA group were collected at the time of knee replacement surgery or other arthroscopic cartilage repair and during the clinical evaluation in PsA and HC groups.

The PG-AG concentration in the HC (healthy control) group was not determined for technical error at the time of laboratory assessment.

### 2.4. Statistical Analysis

Baseline characteristics of participants including age and laboratory findings were presented as means with standard deviations. Distribution of continuous variables was evaluated using the Shapiro-Wilk test. Due to several violations of normality testing to compare more than two groups of the Kruskal-Wallis ANOVA with a suitable post-hoc (Dunn’s) test was used. To identify whether each biomarker could differentiate between patients with OA and PsA univariate and multivariate logistic regression was used (OA = 0, PsA = 1) with age adjustment. In the first step, the regression model containing age and each biomarker as a covariate was calculated. Markers having *p* ≤ 0.2 were entered into a multivariate model with backward elimination [35,36]. The performance of multivariate regression was evaluated using goodness-of-fit statistics (Cox-Snell’s, Nagelkerke’s R^2^). Discriminative ability was assessed by receiver operating characteristic (ROC) curves based on the multivariate models. A *p* value below 0.05 was deemed significant. Analyses were made using Statistica 13 and GraphPad 8 software.

## 3. Results

Finally, 67 people were enrolled in the study: 22 patients with OA, 22 patients with PsA, and 23 individuals who were in the control group of healthy individuals (HC). There were no statistically significant differences between the two groups in terms of age and gender (Table 1).

**Figure 1:**
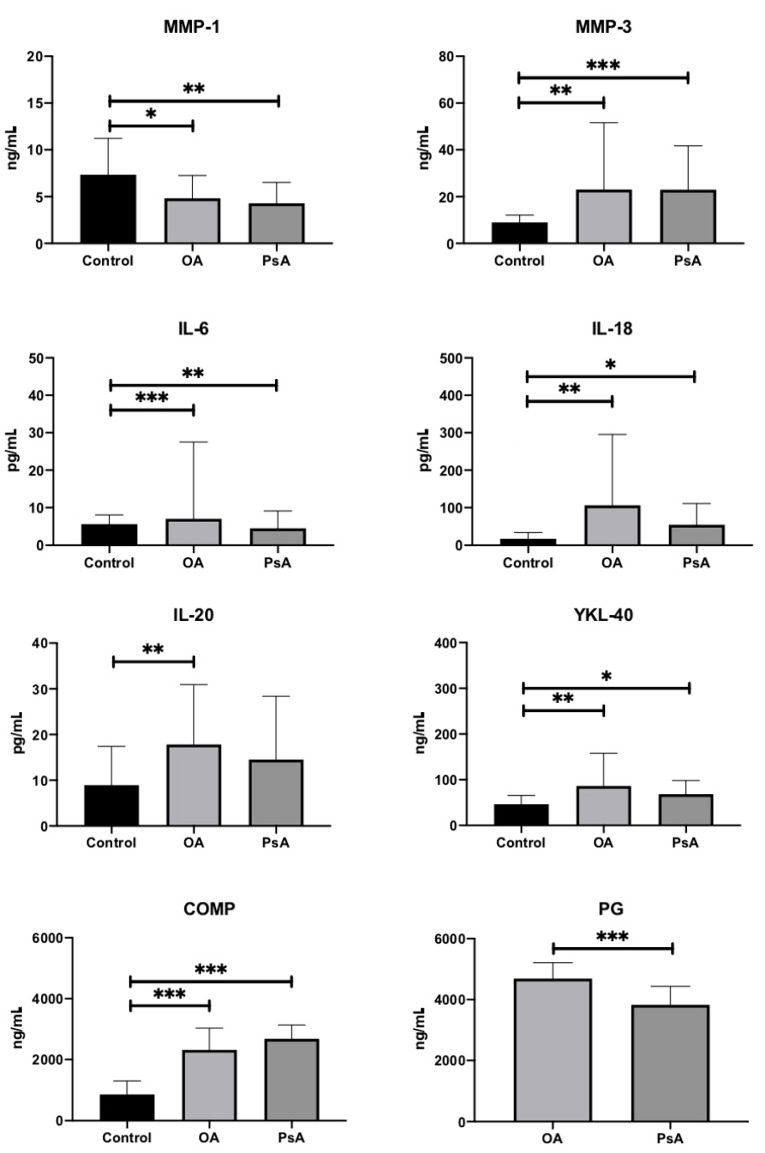
Serum concentration of MMP-1, MMP-3, IL-6, IL-18, IL-20, YKL-40, COMP, and PG-AG in studied groups. Data presented as means (boxes) with standard deviations (whiskers). A comparison between groups was made using Kruskal-Wallis ANOVA (or Mann-Whitney U test) with post-hoc testing indicated above each box-plot. The level of statistical significance indicated with asterisk: * *p* < 0.05, ** *p* < 0.01, *** *p* < 0.001.

In a further analysis, we found a statistically significant increase in the serum concentration of 7 out of 8 examined biomarkers of inflammation and destruction of joint cartilage in the examined patients compared to the control group (Table 1, Figure 1). This significance was observed in both OA and PsA patients (Table 1, Figure 1). The serum concentration of MMP-1 in patients from the control group (HC) was statistically significantly higher than in patients with OA and PsA. Comparing the groups of patients with OA and PsA, the results showed that only the assessment of the PG-AG level in the serum of the examined patients showed statistically significant differences within these two groups of patients (Figure 1). Serum PG-AG level in OA patients was statistically significantly higher than in PsA patients (*p* < 0.001, Figure 1).

**Figure 2:**
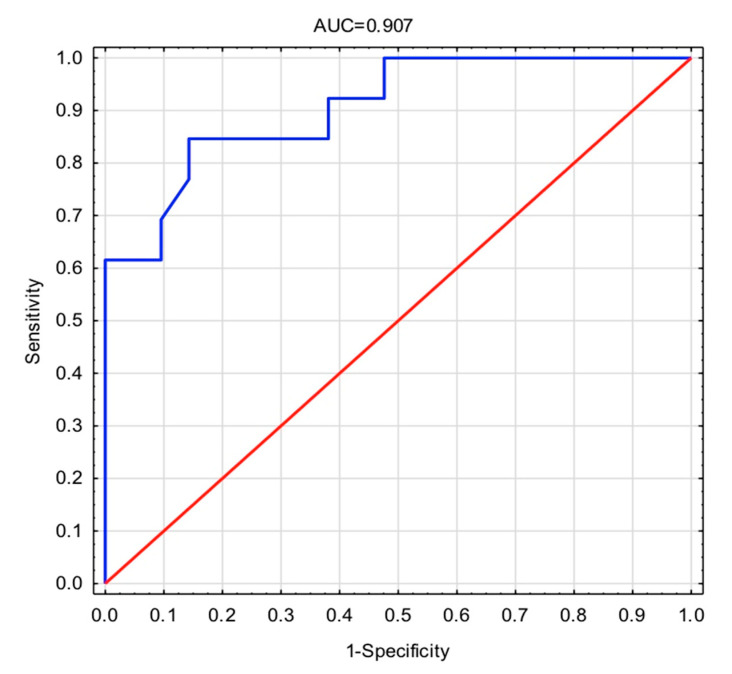
Receiver operating characteristic (ROC) curve of multivariate logistic regression. AUC = 0.907 (for COMP and PG-AG). Red line indicates reference classifier without predictive power. The blue line indicates classifier calculated from multivariate logistic regression for the model including age, COMP and PG-AG.

In our study, we found two markers differentiate patients with OA from patients with PsA. The results of univariate and multivariate logistic regression analysis comparing OA and PsA biomarker serum levels are presented in Table 2. PG-AG and COMP were identified as markers that are significantly different between patients with OA and PsA (odds ratio 0.995 and 1.003, respectively). The ROC curve constructed using the model with age, PG-AG and COMP had an AUC of 0.907 showing that combination of both biomarkers is sensitive and a specific diagnostic tool in differentiation between OA and PsA (Figure 2). Goodness-of-fit statistics (Cox-Snell’s and Nagelkerke’s R^2^) and Hosmer-Lemeshow test (*p* = 0.714) indicate that a constructed model explains more than 45% of the variability of input data (Table 3). Obtained results are suggesting that increased levels of the PG-AG and COMP are associated with the presence of PsA when compared to patients with OA.

## 4. Discussion

For many years, it was believed that the etiology of OA and PsA was completely different. OA are characterized by degenerative processes, whereas PsA are dominated with periarticular inflammatory changes [1,2,3,4,5,6,7]. However, recent years have shown that there are many common features between the two diseases [9,10,11,12,14,37]. Moreover, in many cases, the course of OA is unusual, with inflammatory processes dominating. In some cases, PsA degenerative changes without clear evidence of inflammation dominate. It seems that there are even more factors that make the two diseases similar to each other, and their differentiation may be difficult. In addition to the similar clinical symptoms, certain risk factors are common to OA and PsA. Obesity may be a risk factor for both OA (as is more commonly known) and PsA [38,39,40]. The peak incidence of both of these diseases occurs at the turn of the fifth and sixth decade of life, especially among women, which is related to hormonal changes [4,41]. Post-traumatic damage to periarticular tissues and cartilage may also lead to the development of both OA and PsA [14,42]. The similar symptoms and course of these two diseases may lead to diagnostic problems, delayed treatment, or even ineffective treatment.

Therefore, if the clinical picture, risk factors, and course of the disease in certain situations can make a correct diagnosis difficult, there may be biomarkers that, being specific to OA or PsA, can help differentiate between the two diseases?

In this paper, we tried to answer the question whether there are biomarkers in the serum of OA and PsA patients, which, while participating in inflammatory processes and destruction of articular cartilage, can be both disease-specific and differentiate one from another.

The analysis of the results showed a statistically significant increase in the level of seven out of eight examined markers in the serum of OA and PsA patients as compared to the control group of healthy individuals (Table 1, Figure 1). However, further analysis of the results showed no statistically significant differences in the examined markers of inflammation and destruction of articular cartilage between OA and PsA patients except PG-AG concentration (Table 1, Figure 1).

Our earlier studies confirm the important role these biomarkers play in the etiopathogenesis of both OA and PsA [15,16,17]. Our previous observations in OA patients indicate that IL-18 potentially mediates mainly in intra-articular processes and is responsible for the destruction of joint cartilage. IL-20 could be primarily responsible for the systemic inflammatory reaction [16]. Those observations have also shown that IL-20, but also COMP, YKL-40, and MMP-3, may be a sensitive marker in the diagnosis of osteoarthritis [16]. In turn, our previous studies among patients with PsA indicate the important role of IL-18, COMP, and MMP-3 in the etiopathogenesis of PsA and that these biomarkers may be a sensitive marker of this disease [15,17].

However, no marker considered and analysed separately differentiates one disease (OA) from another (PsA). It was only univariate and multivariate logistic regression analysis comparing OA and PsA biomarker serum levels that has shown that PG-AG and COMP were identified as markers significantly different between patients with OA and PsA (Table 2). The ROC curve constructed using the model with age, PG-AG, and COMP showing that combination of both biomarkers is sensitive and a specific diagnostic tool in differentiation between OA and PsA (Figure 2). Similar observations were made by El-Arman et al. [43]. They also showed statistically significant elevation of COMP and PG-AG levels in serum and synovial fluid (SF )in patients with OA of the knee [43]. In addition, the studies of Chandran at al. indicate that COMP and also MMP-3 show increased serum activity in PsA patients and may be a sensitive marker differentiating PsA and Ps patients from healthy individuals and MMP-3 itself is specific to PsA [44,45]. It seems that previous studies indicate that both PG-AG and COMP clearly reflect changes in joint cartilage of PsA and OA patients [43,44,45]. Moreover, these studies and ours may indicate that the differences in serum concentrations of these biomarkers are too small to be sensitive and specific markers of the diseases are discussed separately.

An interesting observation was also made by Chandran and colleagues in their paper on the participation of selected biomarkers in the pathogenesis of PsA and OA [37]. Their results of multivariate logistic regression analysis comparing PsA and OA serum levels have shown that COMP, resistin, MCP-1, and nerve growth factor (NGF) were identified as markers that were significantly different between patients with PsA and OA [37]. Their ROC curve constructed using the model with age, sex, and the four biomarkers (COMP, resistin, MCP-1, NGF) had an AUC of 0.9984 [37].

Our observations, therefore, confirm the findings from previous studies [14,37] that, sometimes, patients with PsA and OA may form very homogeneous groups, especially in terms of the course of the disease process and its symptoms. This may be reflected in the serum activity of tested biomarkers in OA and PsA patients. The homogeneity of the studied groups indicates that, in case of scanty and uncharacteristic clinical symptoms, the differentiation between the two diseases does not always have to be clear and simple. Our observations, also from previous publications, indicate that the activity of inflammatory processes and destruction of the articular cartilage may be similar in both groups of patients [15,16] and selective consideration of their activity does not allow for differentiation between the two diseases. Only the multivariate logistic regression analysis of the studied biomarkers allowed us to indicate those that distinguish OA from PsA (Table 2).

In our analysis, out of the eight markers studied, only two markers (COMP and PG-AG) proved to be specific and useful in differentiating PsA patients from OA patients. However, only univariate and multivariate logistic regression analysis comparing OA and PsA biomarker serum levels allows us to draw this conclusion. Although the serum concentration of PG-AG in OA patients was statistically significantly higher than in the PsA group, when taken individually, it does not allow for sensitive differentiation between the two diseases. Only a combined analysis of the serum concentrations of PG-AG and COMP in PsA and OA patients, using the model with age, PG-AG, and COMP, allows us to obtain a tool to differentiate between the two diseases. Previous studies indicate that both PG-AG and COMP clearly reflect changes in joint cartilage of PsA and OA patients [43,44,45]. The results from these studies and ours may indicate that the differences in serum concentrations of these biomarkers are too small to be sensitive. Specific markers of the diseases are discussed separately. Recent studies by Chandran et al. also indicate that only a panel of four biomarkers are analyzed together (COMP, resistin, MCP-1, NGF), which can be a sensitive indicator differentiating PsA from OA [37].

Observations from our work and previous ones may also suggest that the overlapping syndrome in PsA may be more frequent than previously thought [17,37,44,45]. It seems possible that there may be more patients with psoriasis and OA in the group of PsA patients who do not respond to biological treatment. Therefore, it seems advisable to continue the research among two groups of PsA patients: responders and non-responders (biological treatment) and to determine their serum biomarkers profile, which could differentiate these two groups of patients. It is possible that this profile of non-responders may be similar to that of OA patients. These assumptions could completely change the approach to study design on evaluation of biomarkers, which differentiate PsA and OA patients.

However, our work has several limitations. The first of them is a relatively small number of patients in groups. These studies, however, are preliminary, but already indicate the advisability of their continuation. The authors of the publication continue their research by extending the group of patients qualified for analysis. It also seems appropriate to analyze the studied biomarkers in serum and synovial fluid simultaneously. This would allow us to draw wider and more objective conclusions. It is also advisable in the future to perform a wider range of immuno-enzymatic studies with determination of activity of other biomarkers involved in inflammatory, cartilage, and bone destruction changes. The other weak point of our study is lack of determination of the serum level of PG-AG in the HC group. The reason for that was a technical error in the laboratory performing our measurements. Due to a lack of sufficient HC serum samples of HC group, we were unable to take new measurements. There is little information and studies available on the evaluation of serum PG-AG levels in OA and PsA patients with respect to the value of this biomarker in healthy volunteers. Data from available studies indicate that there are statistically significant differences between the serum PG-AG levels of OA and PsA patients and healthy people [43,46,47,48].

We are also aware that, although the group of patients with OA was quite homogeneous, mainly with stage 3 and 4 of OA changes, the previous treatment (non-steroid-anti-inflammatory drugs, visco-supplementation) may have influenced the final results. The group of PsA included patients in different stages of the disease with different disease durations. This could also have affected the final results, but, due to the size of the examined group, we could not reliably assess the impact of the duration of symptoms on the serum level of the examined markers.

Undoubtedly, further studies on the markers we have identified, in larger patient groups, are needed to make the results more reliable and objective. This will provide a helpful tool for diagnosing patients with unclear, overlapping images of OA and PsA lesions and may reduce the proportion of PsA patients not responding to biological treatment.

## 5. Conclusions

The results of this study show that COMP and PG-AG may be sensitive markers differentiating patients with osteoarthritis from psoriatic arthritis.

## Figures and Tables

**Table 1:** Demographics, diseases characteristics, and serum biomarker levels in studied groups.

Variable	OA (*n* = 22)	PsA (*n* = 22)	HC (*n* = 23)	*p*-Value *
Age (years)	62.23 ± 12.16	52.82 ± 6.02	54.71 ± 7.40	0.108
Gender (female/male)	11/11	12/10	12/11	0.9554
Disease duration (yeas)	12.9 ± 6.4	14.2 ± 7.2	N/A	-
Kellgren-Lawrence Grading (0–4)	3 (2–4)	N/A	N/A	-
WOMAC score (0–100)	46.42 ± 8.93	N/A	N/A	-
Lequesne index (0–24)	10.31 ± 2.29	N/A	N/A	-
PASI (%)	N/A	16.53 ± 3.93	N/A	-
DLQI	N/A	13.87 ± 2.13	N/A	-
BSA (%)	N/A	26.47 ± 8.41	N/A	-
CRP (mg/L)	N/A	9.48 ± 8.54	N/A	-
TJC + SJC	N/A	10.56 ± 2.53	N/A	-
MMP-1 (ng/mL)	4.83 ± 2.43	4.29 ± 2.23	7.33 ± 3.89	0.003
MMP-3 (ng/mL)	23.06 ± 28.55	22.95 ± 18.72	8.99 ± 3.08	<0.001
PG-AG (ng/mL)	4689.32 ± 518.47	3828.7 ± 603.01	N/A	<0.001
IL-6 (pg/mL)	7.01 ± 20.52	4.48 ± 4.61	5.61 ± 2.45	<0.001
IL-18 (pg/mL)	106 ± 189.76	54.34 ± 56.64	16.73 ± 17	0.001
YKL-40 (ng/mL)	86.23 ± 71.58	68.03 ± 30.21	46.06 ± 19.41	0.003
IL-20 (pg/mL)	17.8 ± 13.13	14.51 ± 13.87	8.9 ± 8.5	0.011
COMP (ng/mL)	2315.61 ± 715.4	2683.91 ± 453.07	862.58 ± 441.31	<0.001

Data presented as means with standard deviations (±SD). Kellgren-Lawrence Grade: the value is given as a median with an interquartile range. * *p*-value refers to the comparison between all three groups using Kruskal-Wallis ANOVA. Detailed results of post-hoc testing are shown in Figure 1. N/A—data not applicable.

**Table 2:** Results of univariate and multivariate logistic regression analyses comparing serum levels of OA to PsA.

Biomarker	Univariate		Multivariate	
	OR (95% CI)	*p*-Value	OR (95% CI)	*p*-Value
MMP-1	0.925 (0.702–1.218)	0.579	-	-
MMP-3	0.999 (0.973–1.025)	0.915	-	-
PG-AG	0.997 (0.995–0.999)	0.007	0.995 (0.991–0.999)	0.023
IL-6	0.982 (0.925–1.043)	0.555	-	-
IL-18	0.997 (0.990–1.004)	0.419	-	-
YKL-40	0.996 (0.984–1.009)	0.561	-	-
IL-20	0.967 (0.911–1.027)	0.277	-	-
COMP	1.001 (1.000–1.002)	0.124	1.003 (1.000–1.005)	0.026

**Table 3:** Multivariate logistic regression goodness-of-fit statistics.

Statistic	Value
−2 Log(Likelihood)	−11.511
Cox-Snell’s R^2^	0.480
Nagelkerke’s R^2^	0.652

## Data Availability

The data used to support the findings of this study will be available with the request to the corresponding author.

## Abbreviations

OA	osteoarthritis
PsA	psoriatic arthritis
HC	healthy control group
IL-18	interleukin-18
IL-20	interleukin-20
IL-6	interleukin-6
MMP-1	metalloproteinase-1
MMP-3	metalloproteinase-3
COMP	cartilage oligomeric matrix protein
PG-AG	aggrecan
YKL-40	human cartilage glycoprotein
DIP	distal interphalangeal joint
PIP	proximal interphalangeal joint
WOMAC	Western Ontario and McMaster Universities Osteoarthritis Index
Lequesne Index	Index of Severity for Osteoarthritis of the Knee
BSA	Body Surface Area
PASI	Psoriasis Area and Severity Index
DLQI	Dermatology Life Quality Index
TJC	the number of tender joints
SJC	the number of swollen joints
MCP-1	monocyte chemoattractant protein-1
NGF	nerve growth factor
ROC	receiver operating characteristic curve

## References

[1] Monibi F., Roller B.L., Stoker A., Garner B.C., Bal S., Cook J.L. (2015). Identification of Synovial Fluid Biomarkers for Knee Osteoarthritis and Correlation with Radiographic Assessment. J. Knee Surg..

[2] Martel-Pelletier J., Barr A.J., Cicuttini F.M., Conaghan P.G., Cooper C., Goldring M.B., Goldring S.R., Jones G., Teichtahl A.J., Pelletier J.-P. (2016). Osteoarthritis. Nat. Rev. Dis. Prim..

[3] Johnson V.L., Hunter D.J. (2014). The epidemiology of osteoarthritis. Best Pr. Res. Clin. Rheumatol..

[4] Gladman D.D., Antoni C., Mease P., Clegg D.O., Nash P. (2005). Psoriatic arthritis: Epidemiology, clinical features, course, and outcome. Ann. Rheum. Dis..

[5] Krajewska-Włodarczyk M., Bechtold A., Żuber Z., Wojtkiewicz M., Wojtkiewicz J. (2019). Role of Microparticles in the Pathogenesis of Inflammatory Joint Diseases. Int. J. Mol. Sci..

[6] Rahimi H., Ritchlin C.T. (2012). Altered Bone Biology in Psoriatic Arthritis. Curr. Rheumatol. Rep..

[7] McGonagle D., Khan M.A., Marzo-Ortega H., Oʼconnor P., Gibbon W., Emery P. (1999). Enthesitis in spondyloarthropathy. Curr. Opin. Rheumatol..

[8] Gottlieb A.B., Mease P.J., Jackson J.M., Eisen D., Xia H.A., Asare C., Stevens S.R. (2006). Clinical characteristics of psoriatic arthritis and psoriasis in dermatologists’ offices. J. Dermatol. Treat..

[9] Tan A.L., Grainger A.J., Tanner S.F., Emery P., McGonagle D. (2006). A high-resolution magnetic resonance imaging study of distal interphalangeal joint arthropathy in psoriatic arthritis and osteoarthritis: Are they the same?. Arthritis Rheum..

[10] McGonagle D., Lories R.J.U., Tan A.L., Benjamin M. (2007). The concept of a “synovio-entheseal complex” and its implications for understanding joint inflammation and damage in psoriatic arthritis and beyond. Arthritis Rheum..

[11] Benjamin M., McGonagle D. (2007). Histopathologic changes at “synovio-entheseal complexes” suggesting a novel mechanism for synovitis in osteoarthritis and spondylarthritis. Arthritis Rheum..

[12] Plato C.C., Norris A.H. (1979). Osteoarthritis of the hand: Age-specific joint-digit prevalence rates1. Am. J. Epidemiol..

[13] Jenkinson T., Bruges-Armas J., Evison G., Cohen M., Lovell C., McHugh N.J. (1994). The cervical spine in psoriatic arthritis: A clinical and radiological study. Rheumatology.

[14] McGonagle D., Hermann K.-G.A., Tan A.L. (2015). Differentiation between osteoarthritis and psoriatic arthritis: Implications for pathogenesis and treatment in the biologic therapy era. Rheumatology.

[15] Waszczykowski M., Bednarski I., Narbutt J., Waszczykowska E., Lesiak A., Fabiś J. (2020). Interleukin-18, interleukin-20, and matrix metalloproteinases (MMP-1, MMP-3) as markers of psoriatic arthritis disease severity and their correlations with biomarkers of inflammation and turnover of joint cartilage. Adv. Dermatol. Allergol..

[16] Waszczykowski M., Fabiś-Strobin A., Bednarski I., Narbutt J., Fabiś J. (2020). Serum and synovial fluid concentrations of interleukin-18 and interleukin-20 in patients with osteoarthritis of the knee and their correlation with other markers of inflammation and turnover of joint cartilage. Arch. Med. Sci..

[17] Waszczykowski M., Bednarski I., Lesiak A., Waszczykowska E., Narbutt J., Fabiś J. (2020). The influence of tumour necrosis factor α inhibitors treatment—Etanercept on serum concentration of biomarkers of inflammation and cartilage turnover in psoriatic arthritis patients. Adv. Dermatol. Allergol..

[18] Wojdasiewicz P., Poniatowski Ł.A., Szukiewicz D. (2014). The Role of Inflammatory and Anti-Inflammatory Cytokines in the Pathogenesis of Osteoarthritis. Mediat. Inflamm..

[19] Nees T.A., Rosshirt N., Zhang J.A., Reiner T., Sorbi R., Tripel E., Walker T., Schiltenwolf M., Hagmann S., Moradi B. (2019). Synovial Cytokines Significantly Correlate with Osteoarthritis-Related Knee Pain and Disability: Inflammatory Mediators of Potential Clinical Relevance. J. Clin. Med..

[20] De Ceuninck F., Sabatini M., Pastoureau P. (2011). Recent progress toward biomarker identification in osteoarthritis. Drug Discov. Today.

[21] Cretu D., Prassas I., Saraon P., Batruch I., Gandhi R., Diamandis E.P., Chandran V. (2014). Identification of psoriatic arthritis mediators in synovial fluid by quantitative mass spectrometry. Clin. Proteom..

[22] Przepiera-Będzak H., Fischer K., Brzosko M. (2016). Extra-Articular Symptoms in Constellation with Selected Serum Cytokines and Disease Activity in Spondyloarthritis. Mediat. Inflamm..

[23] LeQuesne M.G. (1997). The algofunctional indices for hip and knee osteoarthritis. J. Rheumatol..

[24] Bellamy N., Buchanan W.W., Goldsmith C.H., Campbell J., Stitt L.W. (1988). Validation study of WOMAC: A health status instrument for measuring clinically important patient relevant outcomes to antirheumatic drug therapy in patients with osteoarthritis of the hip or knee. J. Rheumatol..

[25] Barr S., Bellamy N., Buchanan W.W., Chalmers A., Ford P.M., Kean W.F., Kraag G.R., Gerecz-Simon E., Campbell J. (1994). A comparative study of signal versus aggregate methods of outcome measurement based on the WOMAC Osteoarthritis Index. Western Ontario and McMaster Universities Osteoarthritis Index. J. Rheumatol..

[26] Wolfe F., Kong S.X. (1999). Rasch analysis of the Western Ontario MacMaster Questionnaire (WOMAC) in 2205 patients with osteoarthritis, rheumatoid arthritis, and fibromyalgia. Ann. Rheum. Dis..

[27] Kellgren J.H., Lawrence J.S. (2008). Radiological Assessment of Osteo-Arthrosis. Ann. Rheum. Dis..

[28] Mease P.J. (2011). Measures of psoriatic arthritis: Tender and Swollen Joint Assessment, Psoriasis Area and Severity Index (PASI), Nail Psoriasis Severity Index (NAPSI), Modified Nail Psoriasis Severity Index (mNAPSI), Mander/Newcastle Enthesitis Index (MEI), Leeds Enthesit. Arthritis Rheum..

[29] Long C.C., Finlay A.Y. (1991). The finger-tip unit—a new practical measure. Clin. Exp. Dermatol..

[30] Ashcroft D.M., Po A.L.W., Williams H.C., Griffiths C.E. (1999). Clinical measures of disease severity and outcome in psoriasis: A critical appraisal of their quality. Br. J. Dermatol..

[31] Finlay A., Khan G. (1994). Dermatology Life Quality Index (DLQI)-a simple practical measure for routine clinical use. Clin. Exp. Dermatol..

[32] Gladman D.D., Mease P.J., Strand V., Healy P., Helliwell P., Fitzgerald O., Gottlieb A.B., Krueger G.G., Nash P., Ritchlin C.T. (2007). Consensus on a core set of domains for psoriatic arthritis. J. Rheumatol..

[33] Altman R., Asch E., Bloch D., Bole G., Borenstein D., Brandt K., Christy W., Cooke T.D., Greenwald R., Hochberg M. (1986). Development of criteria for the classification and reporting of osteoarthritis: Classification of osteoarthritis of the knee. Arthritis Rheum..

[34] Taylor W., Gladman D., Helliwell P., Marchesoni A., Mease P., Mielants H., CASPAR Study Group (2006). Classification criteria for psoriatic arthritis: Development of new criteria from a large international study. Arthritis Rheum..

[35] Wang H., Peng J., Wang B., Lu X., Zheng J.Z., Wang K., Tu X.M., Feng C. (2017). Inconsistency Between Univariate and Multiple Logistic Regressions. Shanghai Arch. Psychiatry..

[36] Bursac Z., Gauss C.H., Williams K., Hosmer D.W. (2008). Purposeful selection of variables in logistic regression. Source Code Biol. Med..

[37] Chandran V., Abji F., Perruccio A.V., Gandhi R., Li S., Cook R.J., Gladman D.D. (2019). Serum-based soluble markers differentiate psoriatic arthritis from osteoarthritis. Ann. Rheum. Dis..

[38] Blagojevic-Bucknall M., Jinks C., Jeffery A., Jordan K. (2010). Risk factors for onset of osteoarthritis of the knee in older adults: A systematic review and meta-analysis. Osteoarthr. Cartil..

[39] Eder L., Abji F., Rosen C.F., Chandran V., Gladman D.D. (2017). The Association Between Obesity and Clinical Features of Psoriatic Arthritis: A Case-control Study. J. Rheumatol..

[40] Cañete J.D., Mease P. (2012). The link between obesity and psoriatic arthritis. Ann. Rheum. Dis..

[41] Oliveria S.A., Felson D.T., Reed J.I., Cirillo P.A., Walker A.M. (1995). Incidence of symptomatic hand, hip, and knee osteoarthritis among patients in a health maintenance organization. Arthritis Rheum..

[42] Olivieri I., Scarpa R., Padula A., D’Angelo S. (2008). Role of Trauma in Psoriatic Arthritis. J. Rheumatol..

[43] El-Arman M.M., El-Fayoumi G., El-Shal E., El-Boghdady I., El-Ghaweet A. (2010). Aggrecan and Cartilage Oligomeric Matrix Protein in Serum and Synovial Fluid of Patients with Knee Osteoarthritis. HSS J..

[44] Chandran V., Cook R.J., Edwin J., Shen H., Pellett F.J., Shanmugarajah S., Rosen C.F., Gladman D.D. (2010). Soluble biomarkers differentiate patients with psoriatic arthritis from those with psoriasis without arthritis. Rheumatology.

[45] Chandran V. (2012). Soluble Biomarkers May Differentiate Psoriasis from Psoriatic Arthritis. J. Rheumatol. Suppl..

[46] Otterness I., Swindell A., Zimmerer R., Poole A., Ionescu M., Weiner E. (2000). An analysis of 14 molecular markers for monitoring osteoarthritis: Segregation of the markers into clusters and distinguishing osteoarthritis at baseline. Osteoarthr. Cartil..

[47] Pedersen S.J., Hetland M.L., Sørensen I.J., Østergaard M., Nielsen H.J., Johansen J.S. (2010). Circulating levels of interleukin-6, vascular endothelial growth factor, YKL-40, matrix metalloproteinase-3, and total aggrecan in spondyloarthritis patients during 3 years of treatment with TNFα inhibitors. Clin. Rheumatol..

[48] Pedersen S.J., Sørensen I.J., Lambert R.G., Hermann K.-G., Garnero P., Johansen J.S., Madsen O.R., Hansen A., Hansen M.S., Thamsborg G. (2011). Radiographic progression is associated with resolution of systemic inflammation in patients with axial spondylarthritis treated with tumor necrosis factor α inhibitors: A study of radiographic progression, inflammation on magnetic resonance imaging, and c. Arthritis Rheum..

